# Single-cell system using monolithic PMUTs-on-CMOS to monitor fluid hydrodynamic properties

**DOI:** 10.1038/s41378-022-00413-y

**Published:** 2022-07-05

**Authors:** Eyglis Ledesma, Iván Zamora, Jesús Yanez, Arantxa Uranga, Núria Barniol

**Affiliations:** Department of Electronics Engineering, Universitat Autònoma de Barcelona, 08193 Bellaterra, Spain

**Keywords:** Electrical and electronic engineering, Engineering

## Abstract

In this work, a single cell capable of monitoring fluid density, viscosity, sound velocity, and compressibility with a compact and small design is presented. The fluid measurement system is formed by a two-port AlScN piezoelectric micromachined ultrasonic transducer (PMUT) with an 80 μm length monolithically fabricated with a 130 nm complementary metal-oxide semiconductor (CMOS) process. The electrode configuration allows the entire system to be implemented in a single device, where one electrode is used as an input and the other as an output. Experimental verification was carried out by exploiting the features of piezoelectric devices such as resonators and acoustic transducers, where a frequency shift and amplitude variation are expected because of a change in density and viscosity. A sensitivity of 482 ± 14 Hz/kg/m^3^ demonstrates the potential of the system compared to other dual-electrode PMUTs. In addition, according to the acoustic measurement, the sound velocity, fluid compressibility, and viscosity coefficient can be extracted, which, to the best of our knowledge, is novel in these PMUT systems.

## Introduction

The characterization of liquid properties is becoming progressively more popular in an increasing number of fields: for example, in the quality assessment in industrial applications, i.e., lubricants^1,2^; in fermentation processes^3^, and in health care applications (i.e., the use of blood density and viscosity changes as an indication of heart disease^4^). In most of these cases, tiny-sized devices that require small amounts of liquid and provide a fast response are desirable, points in which microelectromechanical systems (MEMS) excel. MEMS resonant devices have been widely used as a useful alternative in processes requiring online and in situ monitoring^4–11^

There are three main techniques for extracting liquid parameters with MEMS devices: (a) using resonators and evaluating the change in the resonance frequency and resonator quality factor due to the influence of the liquid surrounding the resonator, which modifies the resonant performance (mass added virtual factor^11–14^); (b) using acoustic devices (SAW devices, quartz microbalances, and FBARs), to measure changes in the frequency response^15,16^ or in a pulse-echo system^17^ due to a change in the acoustic impedance load, which depends on the liquid under test; and (c) using acoustic devices that generate a pressure wave and characterize its propagation and sound attenuation inside the liquid^2,15^. These three techniques allow the characterization of some but not all parameters. For instance, (a) facilitates the characterization of density and viscosity but does not determine the sound velocity or compressibility of the liquid; (b) is limited by surface changes being important for shear viscosity evaluation^2^; and (c) can extract liquid properties such as sound speed and longitudinal viscosity if the density has been previously determined. Numerous examples using the three approaches can be found in the literature. Following approach a), resonant MEMS devices including plates^14^, membranes^12,13^, microcantilevers under different resonant modes such as torsional^18^, microbeam arrays^19^ and suspended channel resonators^9^ have been used as density sensors, using most of them Newtonian fluids with low viscosity of 10 cP. Capacitive and piezoelectric micromachined ultrasound transducers (CMUTs and PMUTs) have also been used as plate flexural resonators for density-viscosity sensing, providing very compact systems on the micrometer scale. In 2016^20^, a system of two CMUTs was used to extract the dynamic viscosity of fluids with high values (from 30 cP to 100 cP) through acoustic measurements. Here, the CMUT was used as an ultrasound device in pulse-echo operation mode, acquiring the time response and computing the FFT to determine the change in resonance frequency, which is a footprint of the liquid damping on the resonator (due to the added virtual mass from the liquid over the resonator^11,13,15^). Unfortunately, the change in mass density was not discussed, and consequently clear interpretation of the cross-sensitivity between density and viscosity was not evaluated.

Other alternatives based on the same operation principle have recently emerged; these methods use piezoelectric micromachined ultrasonic transducers (PMUTs), which require lower driving voltages than CMUTs facilitating their integration into microfluidic systems. One presented in^21^, is a PMUT-fluid-PMUT system with a sensitivity of 292.6 Hz/kg/m^3^ when the PMUT side is 250 μm. However, a pair of PMUTs are needed and, only low viscosity fluids can be measured. To overcome these drawbacks, in^22^, an array is presented where the individual element is a dual-electrode PMUT that facilitates detection of the density change at the expense of decreasing sensitivity (26.3 Hz/kg/m^3^, which is still in the range of the human blood density).

A different approach is followed with CMUT devices in^23^, using an array of independently driven CMUTs that can produce either standing surface waves in the fluid (approach b) or longitudinal acoustic waves (approach c) depending on the array driving. From both kinds of actuation, the system is capable of determining several fluid properties such as density, shear viscosity, and sound velocity, although only shear viscosity at low values is demonstrated experimentally.

In this work, it is demonstrated that a single AlScN PMUT-on-CMOS with two top electrodes could be an excellent alternative, as a minute device capable of determining fundamental mechanical properties of fluids such as density, viscosity, sound velocity, and compressibility. The main contribution of the work is exploiting the integrated system as a resonator or as an acoustic transducer through a pulse-echo system with a combination of approaches a) and c) described above. In this way, a single-cell system can unequivocally sense the density, acoustic viscosity, sound velocity, and compressibility of the fluid being tested, offering added value compared to the state-of-the-art. The single-cell can detect these properties for high density liquids (i.e., Fluorinert (FC-70)), or those with viscosity over 10 cP such as 100 % glycerol, with a density sensitivity of 482 ± 14 Hz/kg/m^3^. Moreover, the presented system is monolithically integrated over a preprocessed CMOS substrate with the adequate circuitry for PMUT driving and sensing. Different from our previous works^24,25^, in this paper AlN doped with Sc is used as the piezoelectric material, providing benefits in terms of piezoelectric transduction coefficients^26^. This single-cell or lab-on-chip for liquid characterization could be easily integrated in a microfluidic cell or hand-held devices of small size, which will make it competitive with respect to other systems ^2,21,22^.

## Materials and methods

**Fig. 1:**
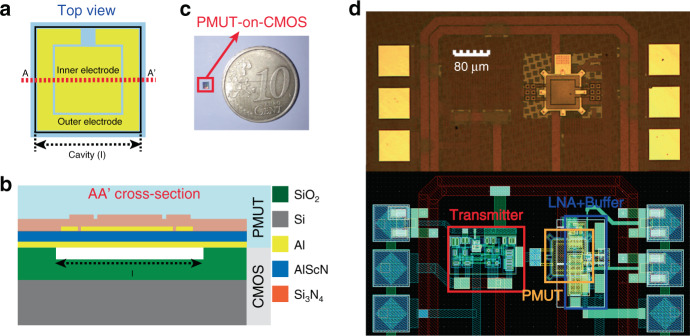
Two-port PMUT device. **a** Schematic PMUT-on-CMOS design top view. **b** AA’ cross-section. (Layers are not to scale). **c** Photograph of the PMUT-on-CMOS devices in comparison with a 10 cents coin. **d** Optical image (top) and schematic layout (bottom) of the PMUT-on-CMOS device. The principal blocks are highlighted in the layout.

The presented PMUT is a two-port device fabricated using the MEMS-on-CMOS process of Silterra^24,27^. As shown in Fig. 1a, b, it consists of a unimorph square structure with an 80 μm side, in which one electrode is used as a transmitter and the other as a receiver. A 0.6 μm AlN with 9.5% Sc piezoelectric layer (Sc_9.5%_Al_90.5%_N) is sandwiched between two top Al electrodes (0.35 μm thick) and one Al bottom electrode (0.4 μm thick). Based on the piezoelectric coefficients (see Table 1 footnote), an improvement in the transduction efficiency is expected compared to that of pure AlN, as shown in^26^. Finally, the PMUT device is covered by 1 μm Si_3_N_4_, which acts as an elastic layer and seals the cavity. The AlScN layer is deposited by physical vapor deposition while the Si_3_N_4_ layer is deposited with low-temperature plasma-enhanced chemical vapor deposition (PECVD) process^28^. All material properties used in theoretical analysis are summarized in Table 1.

**Table 1:** PMUT material properties.

Layer	Material	Young’s modulus (GPa)	Density (kg/m^3^)	Poisson’s ratio
Substrate	SiO_2_	70	2200	0.17
Bottom electrode	Al	70	2700	0.35
Piezoelectric	AlScN^a^	250	3520	0.31
Top electrode	Al	70	2700	0.35
Passive	Si_3_N_4_	250	3100	0.23

^a^ Piezoelectric coefficient for Sc_9.5%_Al_90.5%_N: *e*_31_ = −1.25 C/m^2^ and *e*_33_ = 1.75 C/m^2^ ^26^

The final AlScN PMUT system is monolithically fabricated over the CMOS circuitry integrated into a 130 nm high voltage CMOS^29^, where the outer top electrode is used to generate the acoustic pressure, driven by a high voltage pulser CMOS circuit, while the inner electrode receives the incoming ultrasound wave, which is amplified by a low noise amplifier (LNA)^24,30^. Low voltage switches are used to isolate the transmitter from the receiver. Figure 1d shows an optical image of the PMUT-on-CMOS system presented and its corresponding layout, highlighting the transmitter side (HV pulser), the receiver (LNA + buffer) and the PMUT device.

The natural frequency of a PMUT is defined by Eq. (1), where the value is determined by its physical characteristics: *λ*_*ij*_^*2*^ depends on the vibration mode, the shape and the boundary conditions (*λ*_*ij*_^*2*^ = 35.99 for the first mode corresponding to a square clamped PMUT), *l* is the PMUT side length, *μ* is the mass per unit area, and *D* is the flexural rigidity ^31^.

1 $$f_{{\rm{air}}} = \frac{{\lambda _{ij}^2}}{{2\pi \cdot l^2}}\sqrt {\frac{D}{\mu }}; i = 1,2, \ldots j = 1,2$$

On the other hand, when the PMUT is in contact with a fluid, the resonance frequency is affected by the medium properties, which add extra mass, causing a drop in frequency; see Eq. (2). This parameter is known as added virtual mass (*β*)^13^. It was first determined by Lamb^32^ according to Eq. (3), where the fluid is considered inviscid, and only its density (*ρ*_*liquid*_) causes an increase in *β* and consequently a decrease in the frequency for the same device. The coefficient *Γ* changes depending on the PMUT shape, being 0.342 for a square clamped device^33^:

2 $$f_{{\rm{liquid}}} = \frac{{f_{{\rm{air}}}}}{{\sqrt {1 + \beta } }}$$

3 $$\beta = \Gamma \frac{{\rho _{{\rm{liquid}}} \cdot l}}{\mu }$$

However, viscosity is a relevant property of fluids, so it is important to know the reaction of the transducer at high values of dynamic viscosity (*η*»10 cP). An extension of the Lamb’s model was presented by Kozlovsky in ref. ^34^ where the effect of the viscosity is included in the added virtual mass through ξ; see Eq. (4). Based on Eq. (5), this non-dimensional parameter (*ξ*) depends on the PMUT side length (*l*), the kinetic viscosity (*υ=η/ρ*_liquid_), and the angular frequency (*ω*) in the liquid environment. In addition, Kozlovsky’s model, unlike Lamb’s model, considers Newtonian viscous fluids, allowing quantification of the viscosity contribution to the resonance frequency. In fact, based on Eqs. (4) and (5), a direct relationship between the viscosity and the thickness of the membrane can be extracted ($\xi \propto \left( {fl^2} \right)^{ - 1/2} \propto h^{ - 1/2}$), which shows how the viscosity acquires more importance in thin devices^34^. Furthermore, comparing the added virtual mass for both methods, using Kozlovsky’s model, lower frequencies are reached if the viscosity in the liquid increases.

4 $$\beta = 0.342\frac{{\rho _{{\rm{liquid}}} \cdot l}}{\mu }\left( {1 + 1.057\xi + O\left( {\xi ^3} \right)} \right)$$

5 $$\xi = \sqrt {\frac{\upsilon }{{\omega \cdot l^2}}}$$

In addition to a resonance frequency shift, resonant MEMS devices in liquid suffer from high damping due to the fluid media. This damping is related to the following: (a) the acoustic radiation or mass loading effect, which is proportional to *β* (see Eqs. (6a) and (6b) where *ρ*_*p*_ is the PMUT mass density, *h* is the total PMUT thickness, and *c*_liquid_ is the sound velocity in the liquid), and (b) viscous losses (Eq. (7))^11,13^. Both parameters must be considered in the resonator behavior of the PMUT immersed in a fluid:

6a $$Q_{{\rm{ar}}} = \frac{{\pi \cdot f_{{\rm{liquid}}}}}{\alpha }$$

6b $$\alpha = \frac{{5\pi ^2}}{9}\frac{{ \cdot \rho _{{\rm{liquid}}}}}{{\rho _p}}\frac{{f_{{\rm{liquid}}}^2 \cdot l^2}}{{\left( {1 + \beta } \right) \cdot h \cdot c_{{\rm{liquid}}}}}$$

7 $$Q_{{\rm{vis}}} = \frac{{0.95}}{\xi }\left( {\frac{1}{\beta } + 1} \right)$$

**Fig. 2:**
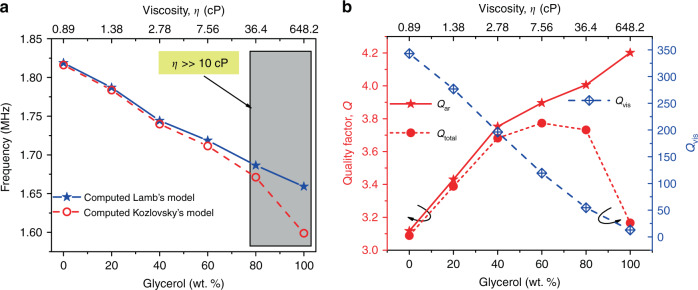
Theoretical analysis of the PMUT at different concentrations of glycerol. **a** Frequency dependence considering Lamb’s model (blue stars) and Kozlovsky’s model (red circles). **b** Contribution of the acoustic radiation (*Q*_ar_ red stars and solid line, left axis) and viscosity (*Q*_vis_ blue squares, right axis) to the global *Q*-factor (*Q*_total_ red points and dash line, left axis).

Considering the PMUT device characteristics, theoretical analysis was carried out using six different water–glycerol mixtures at 29 °C, where the density varies almost linearly throughout the range but the viscosity strongly increases for the last three mixtures from 7.56 cP to 648.2 cP; all properties are summarized in Table 2. As a first step, the resonance frequency for the first flexural mode in air was obtained, which equaled 3.99 MHz. Then, in a liquid environment, the resonance frequency was computed according to both approaches, Lamb and Kozlovsky; see Fig. 2a. The results demonstrate that resonance frequency decreases if the percentage of glycerol increases. Furthermore, according to Kozlovsky’s model, if the viscosity increases (from 80%) the frequency is even lower, demonstrating the effect of the viscosity. Finally, to obtain the PMUT sensitivity to detect the density change, a linear fit was applied (considering Lamb’s model), giving 1.61 kHz/%, which translated in terms of density to 628 Hz/kg/m^3^. Note that the parameter *ξ* must be smaller than 1 to apply Eq. (4), which is fulfilled in our case despite the small size of the PMUT device.

**Table 2:** Water-glycerol mixtures properties.

Property	Glycerol weight percent (%)					
	0	20	40	60	80	100
Density (kg/m^3^)^a^	1000	1045.3	1110	1151.1	1205.5	1254
Viscosity (cP)^b^	0.89	1.38	2.78	7.56	36.4	648.2

^a^Extracted from^14,35^

^b^Computed taking into account the approach presented in ref. ^36^

In relation to the damping, Fig. 2b depicts the quality factor due to the acoustic radiation (Eq. (6a)), the damping due to viscosity (Eq. (7)), and the global Q factor (*Q*_total_^−1^=*Q*_ar_^−1^+*Q*_vis_^−1^). According to the values in Table 2, the acoustic radiation losses are much greater than the viscous losses until viscosity values are above 10 cP. Consequently, for low viscosity liquids, the global resonator *Q*-factor (*Q*_total_) is almost independent of the viscosity. In the case of high viscosity, this term has a clear influence, being difficult to extract the viscosity from the evaluation of the global quality factor due to its reduced value and small variation: the change in the global quality factor, *Q*_total_, ranges from 3.75 to 3.1 (×0.82 factor), while the *Q* for viscosity, *Q*_vis_, changes between 110 to 10 (×0.09 factor) for viscosities between 10 cP and 1000 cP. Because of this, it is not possible to evaluate the effects of the viscosity through the measurement of the PMUT resonator frequency response.

Up to now, the influence of only density and viscosity on the frequency response of the PMUT as a resonator (frequency shift and damping with the evaluation of the quality factor, Q) has been analyzed. Considering the PMUT as an acoustic source in a pulse-echo configuration by changing the travel distance inside the liquid, parameters such as sound velocity and acoustic attenuation can be measured with the time-of-flight and amplitude of the received signal respectively. In relation with the acoustic attenuation and considering the longitudinal or acoustic viscosity, *η*, the damping viscosity coefficient is given by Eq. (8)^15,37,38^,

8 $$\alpha _{p,{\rm{visc}}} \approx \frac{{2 \cdot \pi ^2 \cdot f_{{\rm{liquid}}}^2 \cdot \eta }}{{\rho _{{\rm{liquid}}} \cdot c_{{\rm{liquid}}}^3}}$$

where *f*_liquid_ corresponds to the resonance frequencies in the liquid, *ρ*_liquid_ and *c*_liquid_ are the density and the sound velocity in the liquid environment, respectively. Note the quadratic dependence of this acoustic damping with the frequency.

## Results and discussion

### Fluid characterization using the PMUT as a resonator

**Fig. 3:**
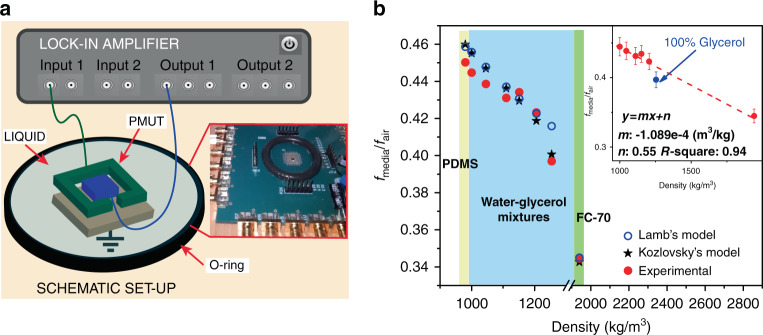
PMUT as a resonator. **a** Experimental setup for the electrical characterization using a lock-in amplifier. Inset: Photograph of the setup. **b** Normalized frequency in different acoustic media considering theoretical (blue circles- black stars) and experimental results (red points). Each material is identified by one color; PDMS: yellow, water–glycerol mixtures: blue, and FC-70: green. Inset: Fitted curve excluding 100% glycerol.

A lock-in amplifier (HF2LI, Zurich Instruments, Switzerland) was used to electrically characterize a simple PMUT device (without any CMOS circuitry connected) wire bonded to a PCB. To find the peak resonance and its amplitude, a frequency sweep was performed according to the theoretical values driving one top electrode with a 10 V continuous wave. The other top electrode was used to detect the frequency change, while the bottom electrode was grounded. Finally, an O-ring with 30 mm diameter was used to confine the liquid over PMUT surface; the setup is shown in Fig. 3a. The fluid test was experimentally performed not only with the water–glycerol mixtures described in Table 2 but also with liquids such as Fluorinert (FC-70) (3 M with ρ_liquid_= 1940 kg/m^3^, η= 24 cP), and elastic materials such as PDMS (10:1, Sylgard 184 Silicone Elastomer with ρ= 980 kg/m^3^) were included.

Several samples were used during the experiment, and before immersing the PMUTs in a liquid environment, they were electrically characterized in air using the same setup. Based on the theoretical value of the frequency for the first mode, a sweep around 4 MHz with a span of 1 MHz was used. A mean resonance frequency of 4.42 MHz with a standard deviation of 0.13 MHz was obtained. Because of this dispersion and to achieve an easy comparison, the resonant frequency (theoretical and measured) in the fluid was normalized with its value in the air (f_media_/f_air_) for the same device. Figure 3b includes the theoretical results considering Lamb’s and Kozlovsky’s models as well as the measured values. As expected, the resonance frequency decreases when the medium density increases. Furthermore, regarding the last point of the water–glycerol mixtures (100% glycerol), the experimental frequency shifts according to Kozlovsky’s model, demonstrating the PMUT’s capability to detect fluids with high viscosity values. Additionally, note how the density plays an important role in the mass loading (β), achieving the highest value when FC-70 is used (lower frequency), even though this sample has 27 times lower viscosity than 100% of glycerol. To evaluate the sensitivity as demonstrated in^22^, the experimental data points were fitted (excluding 100% glycerol due to its high viscosity). The curve fitted using f_media_/f_air_ is a linear function with a slope of −1.089e-4 [1/kg/m^3^] and intercept of 0.55; see Fig. 3b inset. An R-squared value of 0.94 indicates that the presented two-port PMUT can work effectively as a density sensor. According to the mean frequency in the air (f_air_= 4.42 MHz±0.13 MHz), the density sensitivity is 482 ± 14 Hz/kg/m^3^, and the resonance frequency range in the used liquids ranges from 1.5 MHz to 2 MHz. The error here was computed by transferring the dispersion in the electrical measurement in the air (0.13 MHz) to the liquid according to Eq. (2), with a 3% variation from the center frequency.

Table 3 shows a comparison between different PMUTs systems as density sensors. Taking the highest sensitivity^21^, our PMUT achieves 1.6× improvement with a single and compact device. Furthermore, in comparison with other PMUTs, the AlScN PMUT reaches better sensitivity (2.5x that of ref. ^39^ and 18.3x that of ref. ^22^) with a smaller area.

**Table 3:** Comparison of PMUTs as density sensors.

Parameters	2020^39^	2020^21^	2021^22^	This work
Piezoelectric layer	PZT	PZT	PZT	AlScN
TX/RX same chip	Yes	No	Yes	Yes
PMUT size (μm)	500	250	750	80
Sensitivity (Hz/kg/m^3^)	191	292.6	26.3	482

### Fluid characterization using the PMUT as a pulse-echo system

#### Fluid density

Experimental verification was done by immersing the device first in FC-70 and then in 100% glycerol, due to their high density and viscosity, respectively. The air-liquid interface was used as a reflecting surface, and its thickness was adjusted to ensure a time of flight values close to 11 μs in FC-70 and 7 μs in 100% glycerol. To generate the acoustic pressure, the HV transmitter circuit was configured to excite the outer electrode with two cycles of 32 V amplitude.

**Fig. 4:**
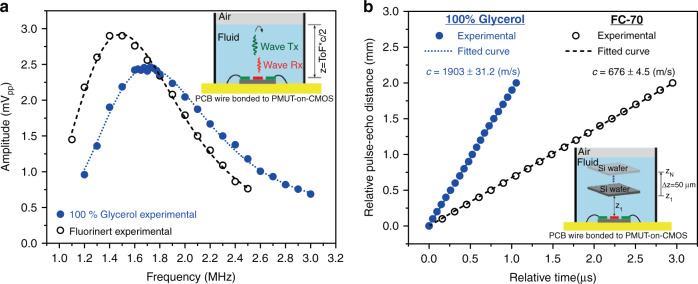
PMUT-on-CMOS as a pulse-echo system: Frequency and sound velocity. **a** Frequency response considering 100% glycerol (blue points) and FC-70 (black circles). The time of flight (ToF) is 7 μs and 11 μs, respectively. **b** Experimental relative distance-time curves to estimate sound velocity in 100% Glycerol (blue point) and FC-70 (black circles). Insets: schematic setups for the frequency response and the sound velocity estimation.

A frequency sweep was carried out in the PMUT in the pulse-echo experiment to identify the resonance frequency (maximum received signal) in each liquid environment. The signal (peak-to-peak amplitude) received by the inner electrode is shown in Fig. 4a. The maximum amplitudes are achieved at 1.49 MHz and 1.71 MHz when FC-70 and 100% glycerol are used, respectively, values close to the electrical measurements (1.54 MHz using FC-70 and 1.81 MHz using 100% glycerol) allowing the determination the density values. In addition, the maximum amplitude for FC-70 is higher than that for 100% glycerol, which is expected for two main reasons: (a) the speed of sound in 100% glycerol is almost 3 times higher, and therefore, the interface liquid-air is further away, and (b) the viscosity coefficient is higher in 100% glycerol than in FC-70 which increases the signal attenuation.

#### Sound velocity

The inset of Fig. 4b shows the schematic setup used to determine the sound velocity in different liquids concentrations. As shown, a piece of silicon wafer (Si-wafer) was used as a reflecting surface, and it was positioned at a base point z_1_. Through a manual micrometer system, the Si-wafer was lifted in increments of 50 μm until it was displaced 1 mm from its original position. Finally, the acoustic pressure was generated by the outer electrode, driven with two cycles with 32 V at the frequency previously determined.

To estimate the sound velocity, cross-correlation was used to obtain the difference in time between the base point and the point of interest. Each time-distance pair was plotted. Figure 4b shows the values, and the slope of a linear fit allows the determination of the sound velocity (*c*), giving 676 ± 4.5 m/s in FC-70 and 1903 ± 31.2 m/s in 100% glycerol in accordance with the reported values^40^. The uncertainty in the sound velocity estimation can be analyzed through the standard deviation (*σ*_*c*_), defined by Eq. (9), where *σ*_*z*_ is the micropositioner accuracy ± 2 μm^41^ and *σ*_*t*_ depends on the sampling frequency (250 MHz for 100% glycerol and 200 MHz for FC-70) giving 4 ns and 5 ns, respectively:

9 $$\sigma _c = \sqrt {\left( {\frac{{{\rm{d}}c}}{{{\rm{d}}z}} \cdot \sigma _z} \right)^2 + \left( {\frac{{{\rm{d}}c}}{{{\rm{d}}t}} \cdot \sigma _t} \right)^2}$$

#### Compressibility

This is an important mechanical property in liquids that indicates a relative change in volume because of a change in pressure, and it can be defined as the inverse of the bulk modulus (*K* = *c*^2^*ρ_liquid_). The fluids being tested, in this case, were the six proposed concentrations of water and glycerol shown in Table 2. The sound velocity was obtained considering the same procedure mentioned above but, unlike the previous section, here, the surface of the PMUT was covered with a 200 μs layer of PDMS (10:1, Sylgard 184 Silicone Elastomer) to isolate the wire bonding and provide good performance during all experiments.

**Fig. 5:**
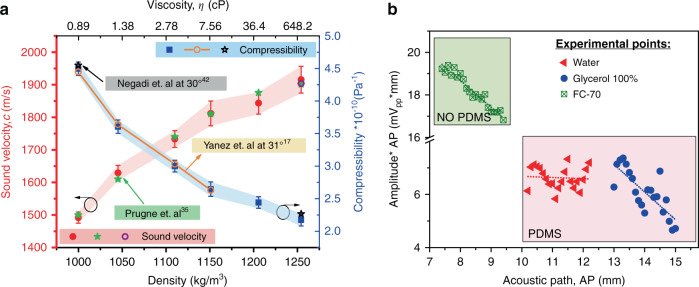
PMUT-on-CMOS as a pulse-echo system: Sound velocity, compressibility, and viscosity. **a** Left axis (red): Sound velocity versus density-viscosity corresponding to water–glycerol mixtures (red points) and data published in ref. ^35^ (green stars); the sound velocity without PDMS is included (purple circle). Right axis (blue): Compressibility estimation and data published in ref. ^17^ (orange circles and line) and in ref. ^42^ (black stars). **b** Pulse-echo amplitude versus acoustic path (AP) with the PMUT immersed in FC-70 (green squares), water (red triangles), and 100% glycerol (blue points). The dotted curves consider the viscosity losses, α_p,visc_, giving 0.021 m^−1^, 4.76 m^−1^, and 1.85 m^−1^ for water, 100% glycerol, and FC-70, respectively.

Figure 5a (left axis) shows the experimental sound velocity obtained (red points) as well as the reported values in^35^ (green stars). The estimated sound velocity without PDMS is also included for 100% glycerol (purple circle), indicating that the PDMS layer does not affect the performance of the PMUT device. On the other hand, the right axis of Fig. 5a shows the compressibility variation for the same density range and its inaccuracy regarding sound velocity (inaccuracy computed as 2(ρ_liquid_*c*^3^)^−1^σ_c_, where *c* is the obtained sound velocity, ρ_liquid_ is the density, and σ_c_ is the standard deviation of the sound velocity). Note that, as expected, an increase in the sound velocity causes lower compressibility values. In addition, based on the reported values in the literature at a temperature close to the one used here (29 °C), the obtained compressibility shows a good correspondence with^17,42^, which demonstrates the high potential of the proposed device.

#### Viscosity

The acoustic losses of the same six water–glycerol mixtures were also studied. To carry out this experiment, the acoustic path (AP) was modified every 100 μm (2*Δ*z*) using the same Si-wafer as a reflecting surface; see the setup in the Fig. 4b inset. Losses as a consequence of liquid damping have been studied in^40,43^ where the propagation waves were considered planar waves and then, only losses caused by the fluid properties affected the signal amplitude. The radiation patterns of small sized PMUTs, such as the one in this work, are almost omnidirectional^44^, and in consequence, the decrease in amplitude is due to the acoustic path (spherical acoustic wave) and the acoustic medium losses due to the viscosity^45^. Considering this, the signal received by the inner electrode is not only reduced by 1/AP but also exponentially decays due to viscous losses (${\rm{e}}^{ - \alpha _{p,{\rm{visc}}} \cdot {\rm{AP}}}$*;* where *α*_*p,*visc_ is defined in Eq. (8)). To see the influence of this second term, the product of the peak-to-peak amplitude (Amp) and acoustic path (AP) was used (Eq. (10)).

10 $${\rm{Amp}}\left( {{\rm{mV}}_{{\rm{pp}}}} \right) \cdot {\rm{AP}}\left( {{\rm{mm}}} \right) = {\rm{e}}^{ - \alpha _{p,{\rm{visc}}} \cdot {\rm{AP}}}$$

Figure 5b shows the experimental points for water (red triangles), 100% glycerol (blue points), and FC-70 (green squares). Measurements were made with the PMUT-on-CMOS covered by 200 μm PDMS (for water and glycerol mixtures) to preserve wire bonding during the experiments. The base point in the acoustic path (*AP*) was determined with AP=2*(*z*_1_ +h_PDMS_) and *z*_1_= (ToF/2- h_PDMS_/c_PDMS_)*c_liquid_ (where ToF = 7 μs is the time of flight, *c*_liquid_ is the sound velocity shown in Fig. 5a, and *h*_PDMS_ = 200 μs and *c*_PDMS_ = 1000 m/s correspond to the thickness and sound velocity of the PDMS). Considering this, the first acoustic path in water is 10.2 mm, and that in 100% glycerol is 13 mm. When FC-70 is used, the PMUT is not covered with PDMS (*h*_PDMS_ = 0 μm), and the ToF is 11 μs, giving an acoustic path of 7.4 mm. To see the influence of viscosity on acoustic losses, an exponential adjustment was performed according to Eq. (10). The coefficient *α*_*p,*visc_ was computed using Eq. (8), giving 0.021 m^−1^, 4.76 m^−1^, and 1.85 m^−1^ for water, 100% glycerol, and FC-70, respectively. These curves are represented by dotted lines on the same graphs. Despite being some dispersion in the experimental points, they show the same trend as the theoretical fit, demonstrating the ability of the PMUT to estimate viscosity.

## Conclusions

In this article, the capabilities of a single AlScN PMUT-on-CMOS for monitoring density, viscosity, sound velocity, and compressibility of fluids are demonstrated. Based on the PMUT behavior, two approaches are presented to characterize different fluids. First, working as a resonator, a change in the liquid density causes a decrease in the resonance frequency, with a sensitivity of 482 ± 14 Hz/kg/m^3^. Second, the propagation of an acoustic wave allows the determination of not only the density but also the sound velocity, which allows the compressibility of the fluid to be characterized. Furthermore, the effect of viscosity is seen in the incoming ultrasonic wave, where the theoretical viscosity coefficient adjusts for the exponential decrease in amplitude. Experimental verification shows that this tiny device, manufactured monolithically on a CMOS substrate, is an excellent candidate for a single measurement cell unit for use in microfluidic systems that require the characterization of the properties of small quantities of fluids. Integrated CMOS circuitry with further signal processing can be easily upgraded to provide smart solutions for demanding industrial and biomedical applications, with constraints on area, power consumption and cost.
